# Complete Genome Sequence of *Geobacillus* sp. Strain E55-1, Isolated from Mine Geyser in Japan

**DOI:** 10.1128/MRA.00339-20

**Published:** 2020-05-14

**Authors:** Kentaro Miyazaki, Erina Hase, Natsuko Tokito

**Affiliations:** a Department of Life Science and Biotechnology, Bioproduction Research Institute, National Institute of Advanced Industrial Science and Technology (AIST), Tsukuba, Ibaraki, Japan; b Department of Computational Biology and Medical Sciences, Graduate School of Frontier Sciences, The University of Tokyo, Kashiwa, Chiba, Japan; Queens College

## Abstract

We report here the complete genome sequence of *Geobacillus* sp. strain E55-1, isolated from the hot sediments of Mine Geyser in Japan. This strain exhibited ∼85% average nucleotide identity and ∼98.5% 16S rRNA sequence identity with the most closely related *Geobacillus* species.

## ANNOUNCEMENT

The genus *Geobacillus* comprises a group of Gram-positive thermophilic bacterial species (1) that were originally classified as group 5 within the genus *Bacillus* but were later reclassified as a new genus, *Geobacillus* (2). Researchers are interested in *Geobacillus* from a biotechnological point of view, particularly as a source of thermophilic enzymes (3).

We collected hot sediment samples at Mine Geyser in Japan (4, 5), which were spread over Lennox-LB agar (1.5% [wt/vol]) plates. After overnight incubation at 55°C, several single colonies were isolated. Colony PCR was conducted to amplify the nearly full-length 16S rRNA gene using a set of primers, Bac8f(C) and UN1541r(U) (6). DNA sequencing analysis suggested that many of them were affiliated with one of the following thermophilic bacilli: *Bacillus*, *Geobacillus*, or *Anoxybacillus*. One of the strains, designated E55-1, showed 98.5% identity with the 16S rRNA genes of a *Geobacillus* species; this strain was subjected to whole-genome analysis by combining Oxford Nanopore Technologies (ONT) and Illumina sequencing technologies. All software analyses were implemented with default settings.

Cells were grown in Lennox-LB broth at 55°C for 18 h. Genomic DNA was extracted from pelleted cells using lysozyme, proteinase K, and achromopeptidase for enhanced cell lysis efficiency (7). For long-read sequencing, genomic DNA was treated with the Short Read Eliminator XS kit (Circulomics). The resulting DNA was used to construct a library using a ligation sequencing kit (SQK-LSK109; ONT). Sequencing was performed using a GridION X5 system with a FLO-MIN106 R9.41 flow cell (ONT) for 6 h. Base calling was performed using Guppy v.3.0.3 (ONT) to generate 68,238 reads corresponding to 781 Mb of genome with an average length of 11,447 bases. The raw reads were filtered (quality [Q], ≧10; read length, ≧1,000 bases) using NanoFilt v.2.3.0 (8). The longest read length was 198,777 bases.

For short-read sequencing, a library with ∼350-bp inserts was generated using the Nextera DNA Flex library prep kit (Illumina), which was subjected to 156-bp paired-end sequencing on the Illumina MiSeq platform. Adapter sequences and low-quality data were trimmed (Q, ≧30; read length, ≧10 bases) using fastp v.0.20.0 (9), yielding 1.03 million paired-end reads, spanning 308 Mb with an average length of 149 bases.

The trimmed long- and short-read data were assembled using Unicycler v.0.4.8 (10) and polished with Pilon v.1.23 (11), generating a single circular chromosome (3,754,053 bp, 51.0 mol% G+C content) and two circular putative plasmids, pGspE55-1 (126,981bp, 45.6 mol% G+C content) and pGspE55-2 (43,187 bp, 43.9 mol% G+C content). Coverages of short reads to the chromosome (72.9×) and plasmid (155.7× for pGspE55-1 and 274.4× for pGspE55-2) sequences suggested a copy number ratio of 1:2:4 in this case.

Automated annotation was performed using DFAST v.1.2.4 (12). The chromosome contained 3,697 coding sequences, 89 tRNA genes, and 26 rRNA genes, while pGspE55-1 and pGspE55-2 contained 134 and 65 coding sequences, respectively. A Web-based JSpeciesWS search (13) revealed that E55-1 showed the highest ∼85% average nucleotide identities with *G. subterraneus* KCTC 3922 (GenBank accession number NZ_CP014342; 14) and *G. kaustophilus* HTA426 (NC_006510; 15), values that were below the cutoff (95%) for novel species (16).

### Data availability.

The complete genome sequences are available from DDBJ/EMBL/GenBank under the accession numbers AP022557 (chromosome), AP022558 (pGspE55-1), and AP022559 (pGspE55-2). Raw sequencing data were deposited in the DDBJ/SRA database under the accession numbers DRX197760 (Illumina MiSeq) and DRX197759 (GridION) (BioProject accession number, PRJDB9279; BioSample accession number, SAMD00204259).
